# Distribution, composition and risk assessment of hydrocarbon residue in surficial sediments of El-Dakhla, El-Kharga and El-Farafra oases, Egypt

**DOI:** 10.1038/s41598-023-46133-9

**Published:** 2023-11-01

**Authors:** Tarek O. Said, Safaa Ragab, Amany El Sikaily, Mohamed A. Hassaan, Ahmed El Nemr

**Affiliations:** Environment Division, National Institute of Oceanography and Fisheries (NIOF), Kayet Bey, El-Anfoushy, Alexandria, Egypt

**Keywords:** Environmental chemistry, Environmental monitoring, Environmental impact

## Abstract

This work examined the polycyclic aromatic hydrocarbons (PAHs) and *n*-alkanes quantities, sources, and hazards in sediments collected from the Egyptian Western Desert Oases namely: Dakhla, Kharga and Farafra oases. The *n*-alkane (C9–C20) residue concentrations have ranged from 0.66 to 2417.91 µg/g recorded for the three Oases. On the other hand, the total *n*-alkane ranged from 448.54 µg/g to 8442.60 µg/g. Higher carbon preference index (CPI) values (> 1.0) proposed that the natural sources could be the main contributor to *n*-alkanes in the Oases sediment. GC-MS/MS (selected reaction monitoring (SRM) method) was used for the determination of the ΣPAHs concentrations in the studied sediments. The ΣPAHs concentrations (ng/g, dry weight) in the studied three Oases varied from 10.18 to 790.14, 10.55 to 667.72, and from 38.27 to 362.77 for the Kharga, Dakhla and Farafra Oases, respectively. The higher molecular weight PAHs were the most abundant compounds in the collected samples. Assessing potential ecological and human health issues highlighted serious dangers for living things and people. All the investigated PAHs had cancer risk values between 1.43 × 10^–4^ and 1.64 × 10^–1^, this finding suggests that PAHs in the samples under study pose a moderate risk of cancer. The main sources of PAHs in this study are biomass, natural gas, and gasoline/diesel burning emissions.

## Introduction

An area of the Sahara known as the Western Desert of Egypt is located west of the Nile River, up to the Libyan border, and south from the Mediterranean to the border with Sudan. Except for a series of oases that stretch from Siwa in the northwest to Kharga in the south of Egypt, the Western Desert is desolate and bleak. In recent years, it has seen fighting, especially during the Second World War^1^. Located in the Western Desert’s geographic heartland, Farafra Oasis is 650 km southwest of Cairo, Egypt. Along the Nile River's two banks, central Egypt's limestone plains extend. It creates an uncomfortable travelling, nearly flat upland desert surface that is only thinly covered in crumbling asphalt consisting of rock and alluvium.

The extraordinary Desert Oases of Dakhla and Kharga are located in the shadow of this limestone level’s southern border, while Farafra and Bahariya Oases may be found north^2^. Farafra is unpredictably triangular in shape and is surrounded by steep-cliffs on three sides^3^. One of the oases in the New Valley Governorate, the Farafra oasis, relies on agricultural operations, which release a significant amount of agricultural drainage water, for its economic survival. This water is collected through drainage ditches in the vacant and uncultivated fields to form a wastewater pond in a highland site. Therefore, the pond’s wall is possibly failing, and the wastewater entering the nearby agricultural areas is present^4^. Sediments in the shallow subsurface may become contaminated by the wastewater flood. Additionally, the population of this region, especially those who are completely reliant on groundwater, faces grave dangers from the leaking of water into the subterranean aquifer. The existence of an impermeable layer, such as shale and clays, stops wastewater from leaking into the freshwater aquifer under the earth^5^.

At Zakhera Village (Dakhla Oasis), in the Western Desert of Egypt, Soltan^6^, performed chemical tests on groundwater and sediment samples on 10 artesian wells. We looked at the interactions between the behaviour of the main and minor ions and the current environmental and geological circumstances. According to him, several factors affect groundwater quality, including hydrogeological conditions, pumping frequency, aquifer depth, sediment type, and human activity, even across small distances. The redox condition of their surroundings and adsorption processes were shown to have a significant impact on the flow coefficient values for metals and non-metals. The saturation index (SI) and water quality index (WQI) indicated the acceptability of these samples for various purposes.

The water quality index (WQI) and saturation index (SI) indicated the acceptability of the sediment samples for various purposes. Almost any historic building remains in the main town, which has all contemporary amenities. An oasis surrounds it, but unlike all the other oases in this region of Egypt, it does not feel like an oasis. In the oasis surrounding the contemporary town of Kharga, there is substantial growth of thorn palm, acacia, buffalo thorn, and jujube. This region is home to several endangered animal species^7^. The fundamental issue with the groundwater aquifer's sustainable growth in Kharga is overexploitation. A significant decline in groundwater heads was seen, reaching 70 to 80 m, the main pattern of water flow was also disrupted, and several exhausted, closed regions were visible. Some groundwater samples have relatively high salinity levels, and abnormally high levels of iron, ranging from 2 to 10 mg/L, which lead to the formation of brown slime and rust-colored deposits on plumbing fixtures, well screens, and pipes. Sodium bicarbonate is the most common form of water, followed by Na_2_SO_4_ and NaCl. Daily groundwater extraction should be reduced for good development and best use of such non-renewable resources; the distance between wells should not be less than 2 km, and the wells’ total depth should be between 600 and 700 m. Additionally, the current flood system of irrigation has to be substituted with more advanced drip and sprinkle irrigation techniques, and less water-intensive crop varieties should be encouraged^8^.

Notably, the Western Desert Oases environment of Egypt has suffered from various pollutants, including persistent organic pollutants (POPs), as a result of the accelerating economic development of the area^2^. Among POPs, PAHs comprise a sizeable subgroup and come from natural and artificial sources^2,9^. In addition to biogenic sources from algae, bacteria, and other marine creatures, the main PAHs sources include oil production, oil spills, incomplete combustion, unintentional grass and forest fires, and fossil fuels. Significant sources of PAHs include air fallout and land-based activities^10^. According to Balmer et al.^11^, PAHs are a diverse family of hydrophobic semi-volatile organic compounds composed of two or more fused aromatic rings and solely include carbon and hydrogen atoms. Due to their carcinogenic, mutagenic, and toxic properties, these organic compounds, which are recognised as priority pollutants by worldwide environmental protection organisations, are of particular interest to geochemists and environmental toxicologists^12^.

Gearhart-Serna et al.^13^, declared that PAHs are considered teratogenic, mutagenic, and highly carcinogenic and may act as endocrine-disrupting chemicals. These organic contaminants may have a long lifetime, resistant to degradation and can cause harmful environmental effects^14^. According to Bai et al.^15^, the four-ring PAH structure through ingestion and dermal-contact routes was the primary contributor to the carcinogenic risk of monomeric PAH in adults or children^12^. There is also an obvious agreement that PAHs may be transferred to be recorded markedly in the human body^16–18^. Thus, a deep interest was motivated to conduct studies on PAHs pollutants found in sediment and their high impact on human health. To estimate the danger of exposure to humans in the Oasis areas of the western desert of Egypt, it is important to look at the residual levels of PAHs in sediment. It is considered the first record (baseline data) of PAHs in Egypt’s Dakhla, Kharga and Farafra Oases.

## Materials and methods

### Materials

The PAHs (13 EPA) investigated in this work were acenaphthylene (ACY), benzo(a)pyrene (BaP), chrysene (CHR), anthracene (ANT), phenanthrene (PHN), benzo(k)fluoranthene (BkF), dibenzo(a,h)anthracene (DBA), benzo(b)fluoranthene (BbF), benzo(a)anthracene (BaA), indeno(1,2,3-cd)pyrene (IP), benzo(g,h,i)perylene (BghiP), fluoranthene (FLR) and pyrene (PYR). GC grade (99.9% pure) n-hexane, CH_2_Cl_2_, and Na_2_SO_4_ anhydrous were all acquired from Merck Millipore. The acetone, CHCl_3_, and CH_3_OH were acquired from Sigma Aldrich in Germany. The authorised multi-component PAH standards combination (EPA 525 PAH Mix A in CH_2_Cl_2_) was delivered by Supelco, USA. Hexane was used to dilute the primary PAH standard, which served as the calibration standard.

### Sample collection

**Figure 1:**
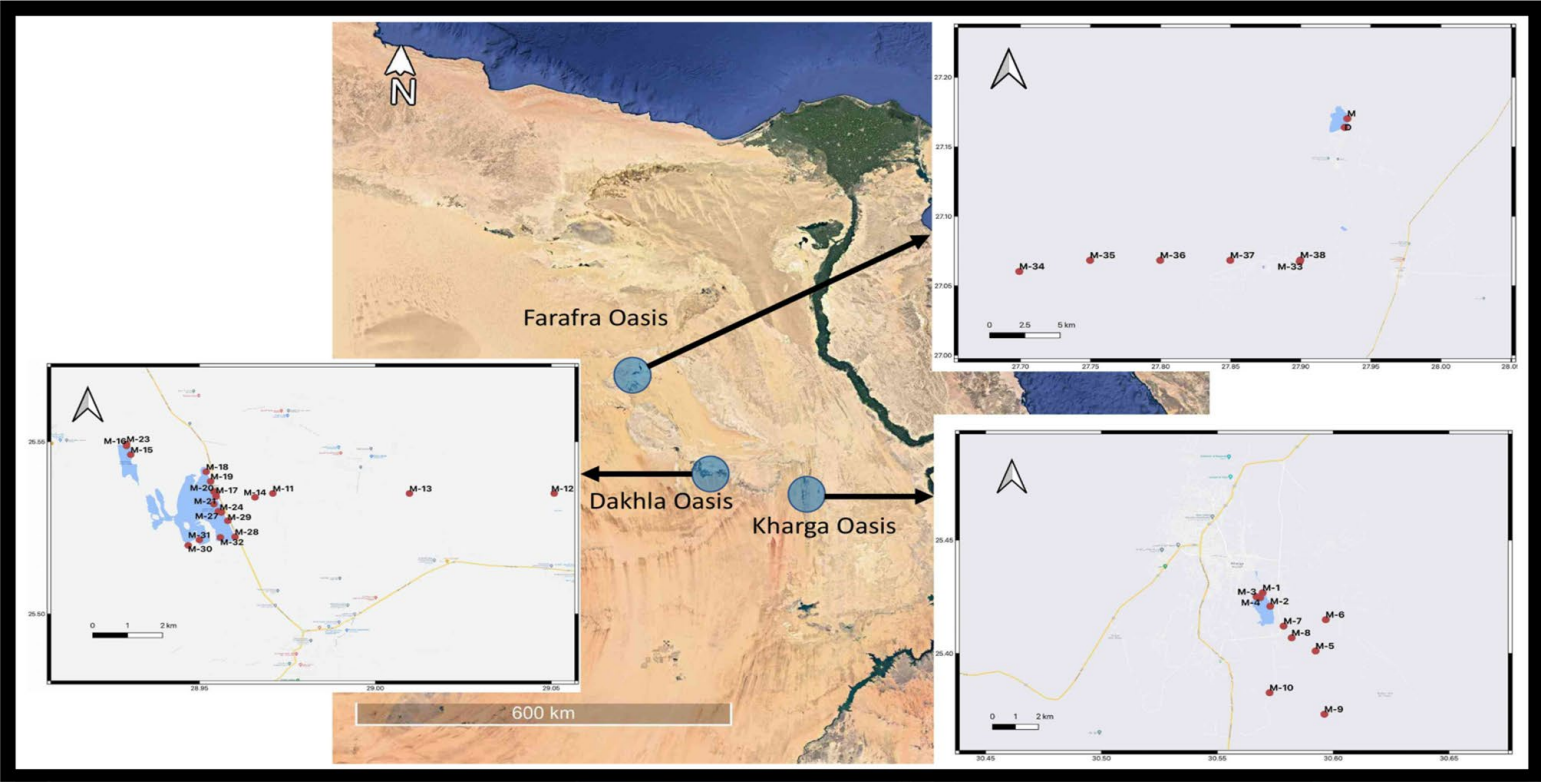
Sampling locations throughout the studied region.

The Western Desert of Egypt contains seven oases and Dakhla Oasis is one of them. It is located between the Farafra and Kharga Oasis in the New Valley Governorate, 350 kilometres from the Nile. It is around 80 km from east to west and 25 km from north to south^5^. Along with several sub-oases, the Dakhla Oasis is home to several villages. Along with a few other minor villages, Mut, El-Masara, and Al-Qasr are the main communities. In all, 40 surface sediment samples were taken from three Oases in Egypt’s New Valley Governorate (Fig. 1) by the mean of the van Veen grab sampler. The areas were chosen while considering the anticipated pollution brought on by human activity (Table S1 in the Supplementary Material). A global positioning system (GPS) was applied to get the coordinates of the sampling sites. The sediments were gathered using a stainless steel grab. Each site received six grabs, and the top 3 cm were scooped into clean, wide-mouth glass bottles, transported frozen, and stored at 20 °C until analysis. Each sample was gathered and weighed at a weight of around 5 g in an aluminium dish. The C-9 to C-20 *n*-Alkanes and 13 EPA PAHs were analysed in the sediment samples.

### Sample pre-treatments

Following a dichloromethane-hexane extraction, sediment heterogeneity in terms of the PAHs distribution was first investigated to look into variations in PAH contents between replicate sediment samples. The findings revealed that the pollutants were evenly distributed throughout the soils, with no variation in total PAH amounts across replicate soil samples (data not shown). The sample was extracted with the use of ultrasound. Individual samples were taken out of the refrigerator, defrosted at ambient temperature for about five hours, and then dried at 50 °C for a whole night before being subjected to chemical treatment. Then, 5 g of anhydrous sodium sulphate was well mixed with 5.0 g of each sample. Each sediment sample was used to make duplicates. Next, the sediment sample was sonicated in an ultrasonic bath with 2 × 100 mL *n*-Hexane for 30 min each, and a third extraction with CH_2_Cl_2_ (100 mL) was performed. The three extracts were then mixed, desulfurized using Cu metal-activated powder, and concentrated to a few millilitres at around 35 °C in a rotary evaporator. Finally, they were concentrated using a N_2_ gas down to approximately 1 mL. Cleaning and fractionation were accomplished by putting the concentrated extract through a column made of silica and Al_2_O_3_. The column was made ready by slurry packing 20 mL (10 g) of silica, 10 mL (10 g) of Al_2_O_3_, and then 1.0 g of anhydrous Na_2_SO_4_. For the saturated aliphatic fraction (F1), the extract (1 mL) was successively eluted from the column with 25 mL of *n*-Hexane. Then 70 mL of dichloromethane and n-hexane (80:20) were added for the PAH fraction (F2). To prepare F1 and F2 for further chromatographic examination, a gentle stream of pure N2 was used to concentrate them. Since hexane is a nonpolar solvent, it was utilised as the injection solvent to enhance fraction separation.

### Instrumental analysis

Because of the excellent specificity and sensitivity of this analytical method for contaminated soil samples, individual PAHs were identified and quantified using GC-MS/MS. A DB5 ms ultra-inert capillary column, with an internal diameter of 0.25 mm, length of 30 m, and thickness of 0.25 m, was used to separate the samples. Splitless mode and a 1 mL injection volume were used for the analysis. Detection of the analytes was performed by employing a Thermo TRACE™ 1300 gas chromatography equipped with PTV mode splitless injector (Temperature 80 °C, Splitless time 1 min, Purge flow 5 mL/min, Carrier gas saver flow 20 mL/min for 5 min, Transfer temperature delay 1 min, Injection pressure 70 kPa for 0.1 min, Transfer pressure 210 kPa, Transfer rate 10 °C/sec, Transfer temperature 300 °C, Transfer time 3 min, Cleaning rate 10 °C/sec, Cleaning temperature 320 °C, and Cleaning time 10 min) coupled to a TSQ 8000 Evo mass spectrometer (Thermo, USA) operating in selected reaction monitoring (SRM) mode and the Ar gas collision cell was set at 1.5 mL/min. Thermo TriPlus RSH Autosampler was used^19–22^.

The transfer line was set to 300 °C, the ion source temperature was set to 270 °C, the total scan duration was set to 0.1 sec, the predicted chromatographic peak width was set to 2.0 sec, and the timed scan type was set to SRM for the study of PAHs. The integrated peak area ratio of the target ion to the external standard was used to quantify the analytes. Target ions and retention time order allowed for the identification of the PAH analytes. Table S2 (Supplementary Material) displays the SRM and EI energies applied to PAHs. The GC oven temperature program used for PAH analysis starts at 60 °C and holds that temperature for 1 minute before increasing to 140 °C at a rate of 20.0 °C/min and holding that temperature for 0.0 min before increasing to 300 °C at a rate of 5.0 °C/min and holding that temperature for 4 mins. Thermo Scientific Xcalibur was used for data capture, reprocessing, and report production.

The transfer line was set to 300 °C, the ion source temperature was set to 270 °C, the total scan duration was set to 0.1 sec, the predicted chromatographic peak width was set to 2.0 sec, and the timed scan type was set to SRM for the study of *n*-alkanes. The integrated peak area ratio of the target ion to the external standard was used to quantify the n-alkanes. Target ions and retention time order were used to identify the *n*-alkanes. For *n*-alkanes, the SRM and EI energies are displayed in Table S3 (Supplementary material). The GC oven temperature program used for n-alkanes analysis starts at 65 °C and holds that temperature for 2 minutes before increasing to 250 °C at a rate of 15.0 °C/min and holding that temperature for 0.0 mins before increasing to 300 °C at a rate of 8.0 °C/min and holding that temperature for 2 mins. Thermo Scientific Xcalibur was used for data capture, reprocessing, and report production.

Quality control (QC) and quality assurance (QA) techniques were used to guarantee the correctness and precision of the analytical results. Blank analysis, duplicate extract analysis, and reanalysis of samples with relative percent differences greater than 20% were performed^23,24^. Additionally, standard curves were calibrated daily using reference standards, and calibration levels were checked after every ten extract analyses. The analyte amounts in the blank samples were insignificant or below the limit of detection (LOD). The TSQ instrument’s regression coefficient for the calibration curves of analytes varied from 0.975 to 0.997. Each method’s results were extremely accurate, with a relative error range of 0.1 to 5.0%, and the findings of each approach were quite accurate. The recovery rates of n-alkanes and PAH residues from water and sediment samples ranged from 92.4 to 107.8%.

## Results and discussions

### Physiochemical parameters

Table S1 (in Supplementary Material) displays the results of physicochemical parameters for the water body of the sampling sites of the collected surficial sediment. The water's average Temp., pH, EC and TDS were 19.6 °C, 8.12, 41364.9 μs/cm and 26490.5 ppm, respectively.

### Composition, distribution, and concentration of PAHs

Tables S4a–c (Supplementary Material) show each PAH's residue levels in all tested samples. The ∑PAHs concentrations varied significantly, ranging from 10.18 to 790.14 ng/g with a mean value of 166.44 ng/g. The average concentrations of ∑PAHs were recorded in the collected sediment samples in the following descending order: Farafra > Dakhla > Kharga. In all of the analysed samples, PHN was the predominant congener. The order was: PHN 158.89 > PYR 137.24 > BaP 109.19 > CHR 96.69 > BaA 84.59 > FLU 83.32 > BbF 72.27 > DahA 70.02 > BghiP 55.71 > ANT 53.30 > BkF 43.66 > ACY 42.92 > IP 17.08 ng/g. The current study's order demonstrates that HMW PAHs predominated. This could be explained by HMW PAHs in bottom sediments having a lower solubility due to their adsorption on particulate matter^16^.

**Figure 2:**
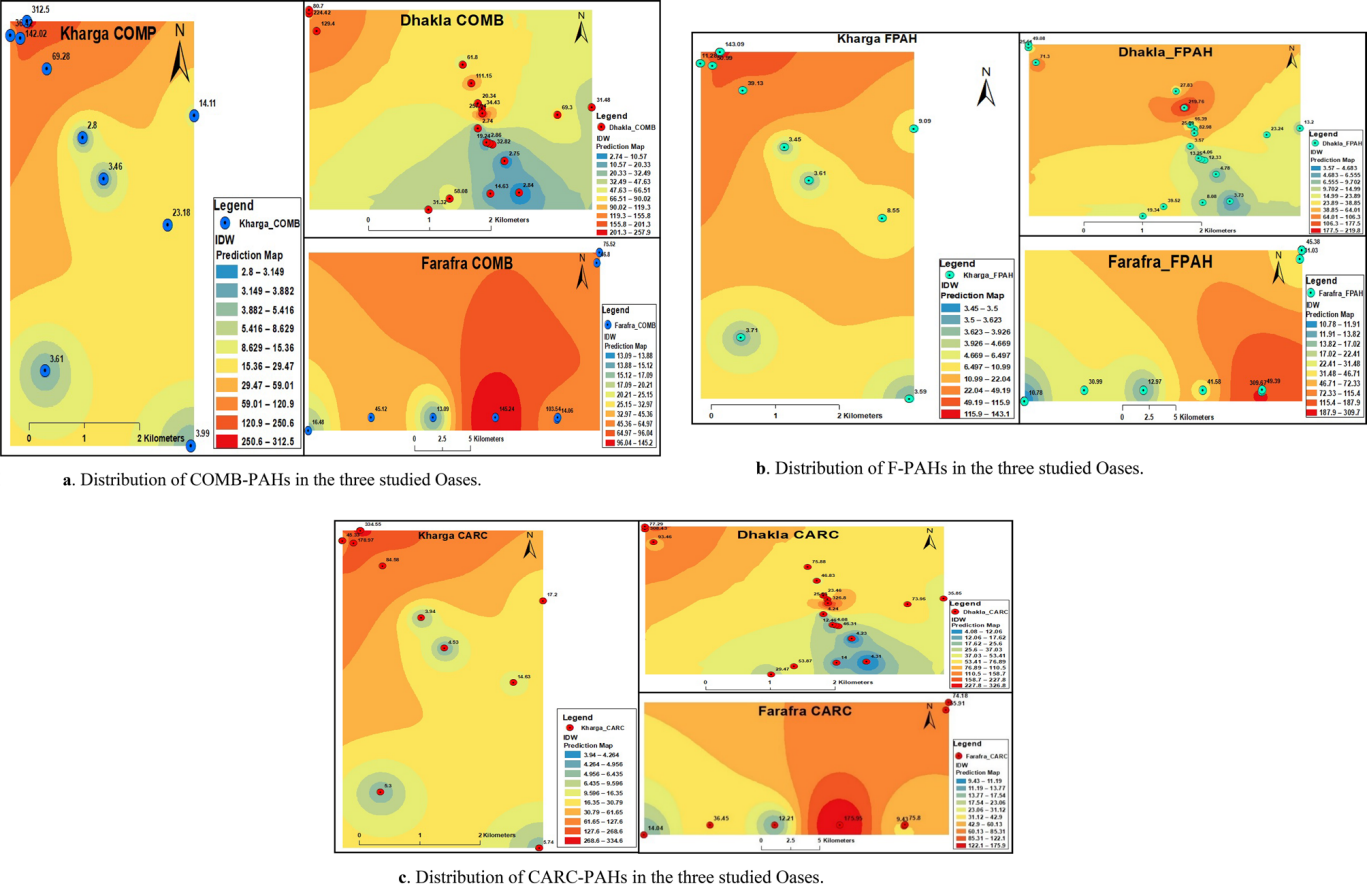
(**a**) Distribution of COMB-PAHs in the three studied Oases. (**b**) Distribution of F-PAHs in the three studied Oases. (**c**) Distribution of CARC-PAHs in the three studied Oases.

**Figure 3:**
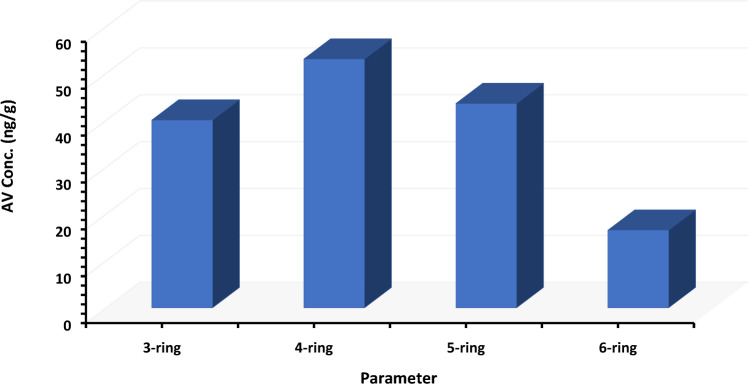
Distribution of the number of ring structures of PAHs in the collected samples.

The ∑PAHs, %, and ratios are presented in Tables 1, 2 and 3. For ∑PAHs, ∑PAH_CARC_ (BaA +BbF + BaP + DahA + IP) were well-thought-out carcinogens^25^. The highest concentration level of ∑PAH_CARC_ was documented in Kharga (69.48 ng/g), followed by Dakhla (65.04 ng/g). ∑PAH_CARC_ ranged from 94-334.55 ng/g with 37.58% of total PAHs. Moreover, the average of ∑PAH_COMB_ (PYR + CHR + BkF + BghiP) ranged from 61.14 to 63.85 ng/g, with an average of 35.44% of total PAHs. Additionally, ∑PAH_F_ varied between 22.65 to 32.50 ng/g with 26.98% of total PAHs. Figure 2 indicates the descending order of various groups of PAHs from CARC > COMB > FPAHs. Figure 3 represents the distribution of ring structures of PAHs. The descending order was obviously 4-ring > 3-ring > 5-ring > 6-ring PAHs with average concentrations of 43.40, 52.61, 43.10 and 15.81 ng/g, respectively. Petroleum products or crude oil are the principal sources of petrogenic pollutant-derived PAHs, with three- to four-ring structures. While pyrolytic pollutant-produced PAHs have 4-6 rings built into them^26^. Generally, PAHs containing > 4-rings are believed to be more toxic than PAHs, which have two or three aromatic rings^27^. On the other hand, the contribution of potential pollution sources was identified and quantified using the diagnostic ratios of the residue levels of different PAHs^28,29^. The PHN/ANT ratios in this study’s samples were all less than 10 (Tables 1, 2 and 3), confirming pyrolytic origin. Notably, the BaA/CHR isomeric ratio was close to 1, which supports the comparable source.

**Table 1:** Interrelationship indices of PAHs recorded in Kharga Oasis (ng/g, dry weight (dw)).

Analysis data	Kharga Oasis										Av	Min	Max
	M1	M2	M3	M4	M5	M6	M7	M8	M9	M10			
∑PAHs	790.14	192.99	93.03	371.98	46.36	40.40	10.18	11.60	13.32	12.62	158.26	10.18	790.14
∑CARC^1^	334.55	84.58	45.33	178.97	14.63	17.20	3.94	4.53	5.74	5.30	69.48	3.94	334.55
∑CARC%	42.34	43.83	48.73	48.11	31.55	42.58	38.66	39.06	43.09	41.98	41.99	31.55	48.73
∑COMB^2^	312.50	69.28	36.42	142.02	23.18	14.11	2.80	3.46	3.99	3.61	61.14	2.80	312.50
∑COMB%	39.55	35.90	39.14	38.18	50.01	34.93	27.47	29.86	29.92	28.61	35.36	27.47	50.01
∑FPAHs^3^	143.09	39.13	11.28	50.99	8.55	9.09	3.45	3.61	3.59	3.71	27.65	3.45	143.09
∑FPAHs%	18.11	20.28	12.13	13.71	18.44	22.49	33.87	31.08	26.98	29.41	22.65	12.13	33.87
∑3-rings	143.09	39.13	11.28	50.99	8.55	9.09	3.45	3.61	3.59	3.71	27.65	3.45	143.09
∑4-rings	301.65	69.74	24.11	131.64	10.86	8.77	2.31	2.76	2.81	2.65	55.73	2.31	301.65
∑5-rings	209.36	51.12	32.10	108.48	10.20	12.09	2.49	2.82	3.03	3.00	43.47	2.49	209.36
∑6-rings	66.01	17.39	13.91	41.18	12.94	6.35	1.20	1.53	2.59	2.05	16.51	1.20	66.01
LMW PAHs^4^	143.09	39.13	11.28	50.99	8.55	9.09	3.45	3.61	3.59	3.71	27.65	3.45	143.09
HMW PAHs^5^	577.03	138.24	70.11	281.29	34.00	27.20	6.00	7.11	8.42	7.70	115.71	6.00	577.03
LWM/HMW	0.25	0.28	0.16	0.18	0.25	0.33	0.58	0.51	0.43	0.48	0.34	0.16	0.58
ANT/(ANT + PHE)	0.20	0.26	0.14	0.24	0.41	0.33	0.52	0.54	0.47	0.55	0.37	0.14	0.55
FLU/(FLU + PYR)	0.09	0.13	0.27	0.13	0.33	0.51	0.53	0.44	0.43	0.47	0.33	0.09	0.53
IP/(IP + BghiP)	0.22	0.26	0.19	0.21	0.09	0.17	0.57	0.53	0.55	0.56	0.33	0.09	0.57
PHN/ANT	3.90	2.84	5.99	3.14	1.47	1.99	0.93	0.87	1.12	0.83	2.31	0.83	5.99
BaA/CHR	1.06	1.12	1.14	1.07	0.82	1.19	1.23	1.19	1.20	1.24	1.13	0.82	1.24
BaP/BghiP	2.11	2.00	0.95	1.66	0.23	0.48	1.85	1.49	0.96	1.31	1.30	0.23	2.11
BaP/BbF	1.93	1.47	0.85	1.43	0.55	0.41	1.17	1.10	1.04	1.26	1.12	0.41	1.93
BaP/BkF	2.50	3.35	1.20	3.17	1.03	0.76	1.36	1.36	1.28	1.38	2.42	1.36	2.50
IP/BghiP	0.28	0.34	0.24	0.27	0.09	0.21	1.31	1.13	1.24	1.26	0.64	0.09	1.31

^1^Carcinogenic PAHs, ^2^Combustion PAHs, ^3^fossil-fuel PAHs, ^4^Low molecular weight PAHs, ^5^High molecular weight PAHs.

**Table 2:** Interrelationship indices of PAHs recorded in Dakhla Oasis (ng/g, dry weight (dw)).

Analysis data	Dakhla Oasis																						Av	Min	Max
	M11	M12	M13	M14	M15	M16	M17	M18	M19	M20	M21	M22	M23	M24	M25	M26	M27	M28	M29	M30	M31	M32			
∑PAHs	80.53	239.44	237.48	166.49	294.17	183.50	85.84	165.51	377.74	60.19	667.72	91.46	581.93	44.94	40.02	10.55	11.00	10.89	11.77	80.12	151.47	36.70	164.98	10.55	667.72
∑CARC	35.85	71.42	84.92	73.95	93.46	77.29	25.53	75.88	46.83	23.46	326.80	46.31	308.43	12.46	14.19	4.24	4.08	4.31	4.23	29.47	53.87	14.00	65.04	4.08	326.80
∑CARC%	44.52	29.83	35.76	44.41	31.77	42.12	29.74	45.85	12.40	38.98	48.94	50.63	53.00	27.72	35.45	40.16	37.07	39.60	35.96	36.78	35.56	38.14	37.93	12.40	53.00
∑COMB	31.48	100.78	104.34	69.30	129.40	80.70	34.43	61.80	111.15	20.34	257.94	32.82	224.42	19.24	11.36	2.74	2.86	2.84	2.75	31.32	58.08	14.63	63.85	2.74	257.94
∑COMB%	39.09	42.09	43.94	41.63	43.99	43.98	40.11	37.34	29.42	33.79	38.63	35.89	38.56	42.81	28.38	25.98	25.99	26.12	23.39	39.09	38.34	39.85	36.29	23.39	43.99
∑FPAHs	13.20	67.23	48.22	23.24	71.30	25.51	25.89	27.83	219.76	16.39	82.98	12.33	49.08	13.25	14.47	3.57	4.06	3.73	4.78	19.34	39.52	8.08	36.08	3.57	219.76
∑FPAHs%	16.39	28.08	20.31	13.96	24.24	13.90	30.16	16.81	58.18	27.23	12.43	13.48	8.43	29.47	36.17	33.86	36.94	34.29	40.65	24.13	26.09	22.01	25.78	8.43	58.18
∑3-rings	13.20	67.23	48.22	23.24	71.30	25.51	25.89	27.83	219.76	16.39	82.98	12.33	49.08	13.25	14.47	3.57	4.06	3.73	4.78	19.34	39.52	8.08	36.08	3.57	219.76
∑4-rings	27.75	85.50	93.33	46.91	94.87	66.10	26.34	52.85	99.14	13.01	239.96	26.85	193.72	15.97	8.48	2.15	2.43	2.21	2.33	21.29	46.80	8.44	53.47	2.15	239.96
∑5-rings	22.37	63.24	69.71	53.33	63.74	56.45	19.44	48.64	33.33	17.01	206.87	29.04	198.11	8.70	9.78	2.60	2.54	2.68	2.71	20.14	37.74	9.19	44.43	2.54	206.87
∑6-rings	9.27	22.62	25.71	24.54	43.70	20.00	8.71	19.88	9.82	7.80	69.21	11.04	71.81	4.54	4.37	1.35	1.21	1.47	1.20	11.44	15.30	7.33	17.83	1.20	71.81
LMW PAHs	13.20	67.23	48.22	23.24	71.30	25.51	25.89	27.83	219.76	16.39	82.98	12.33	49.08	13.25	14.47	3.57	4.06	3.73	4.78	19.34	39.52	8.08	36.08	3.57	219.76
HMW PAHs	59.39	171.36	188.74	124.78	202.31	142.55	54.49	121.38	142.30	37.82	516.04	66.93	463.65	29.21	22.64	6.10	6.18	6.36	6.24	52.88	99.84	24.96	115.73	6.10	516.04
LWM/HMW	0.22	0.39	0.26	0.19	0.35	0.18	0.48	0.23	1.54	0.43	0.16	0.18	0.11	0.45	0.64	0.59	0.66	0.59	0.77	0.37	0.40	0.32	0.43	0.11	1.54
ANT/(ANT + PHE)	0.11	0.20	0.29	0.26	0.16	0.19	0.11	0.23	0.31	0.15	0.20	0.23	0.21	0.16	0.16	0.57	0.53	0.57	0.48	0.13	0.13	0.35	0.26	0.11	0.57
FLU/(FLU + PYR)	0.20	0.28	0.22	0.27	0.15	0.13	0.33	0.24	0.99	0.58	0.07	0.24	0.11	0.39	0.70	0.63	0.56	0.60	0.69	0.34	0.36	0.43	0.39	0.07	0.99
IP/(IP + BghiP)	0.28	0.07	0.08	0.19	0.13	0.20	0.17	0.22	0.50	0.19	0.25	0.27	0.22	0.24	0.27	0.48	0.55	0.52	0.54	0.18	0.20	0.18	0.27	0.07	0.55
PHN/ANT	8.14	3.97	2.39	2.81	5.23	4.19	8.05	3.32	2.20	5.76	3.94	3.39	3.74	5.30	5.16	0.74	0.87	0.76	1.09	6.71	6.53	1.86	3.92	0.74	8.14
BaA/CHR	0.80	0.74	0.97	0.68	0.81	0.79	0.57	0.79	0.02	0.95	0.99	0.81	1.04	0.55	1.14	1.19	1.18	1.20	1.21	0.64	0.87	0.83	0.85	0.02	1.21
BaP/BghiP	1.46	1.10	1.24	0.94	0.63	1.38	0.45	1.33	4.79	0.74	1.97	1.62	1.73	0.50	0.68	1.47	1.79	1.48	1.96	0.87	1.11	0.54	1.35	0.45	4.79
BaP/BbF	1.19	0.87	1.02	0.92	1.09	1.11	0.31	1.02	45.52	0.62	1.42	1.14	1.43	0.38	0.45	1.18	1.12	1.13	1.17	1.20	0.96	0.92	1.20	1.90	1.42
BaP/BkF	2.20	1.77	2.58	1.30	1.37	1.50	0.60	2.68	2.59	1.01	3.23	2.77	2.78	0.72	0.81	1.48	1.45	1.45	1.57	1.55	1.37	1.40	2.13	1.45	2.97
IP/BghiP	0.38	0.07	0.09	0.23	0.15	0.25	0.20	0.28	0.99	0.23	0.33	0.38	0.29	0.31	0.37	0.93	1.20	1.08	1.16	0.23	0.24	0.21	0.44	0.07	1.20

**Table 3:** Interrelationship indices of PAHs recorded in Farafra Oasis (ng/g, dry weight (dw)).

Analysis data	Farafra Oasis								Av	Min	Max
	D	M	M33	M34	M35	M36	M37	M38			
∑PAHs	333.16	41.30	112.57	38.27	362.77	228.72	133.73	195.08	180.70	38.27	362.77
∑CARC	9.43	14.04	36.45	12.21	175.95	75.80	55.91	74.18	56.75	9.43	175.95
∑CARC%	2.83	33.99	32.38	31.91	48.50	33.14	41.81	38.03	32.82	2.83	48.50
∑COMB	14.06	16.48	45.12	13.09	145.24	103.54	46.80	75.52	57.48	13.09	145.24
∑COMB%	4.22	39.91	40.08	34.20	40.04	45.27	34.99	38.71	34.68	4.22	45.27
∑FPAHs	309.67	10.78	30.99	12.97	41.58	49.39	31.03	45.38	66.47	10.78	309.67
∑FPAHs%	92.95	26.10	27.53	33.89	11.46	21.59	23.20	23.26	32.50	11.46	92.95
∑3-rings	309.67	10.78	30.99	12.97	41.58	49.39	31.03	45.38	66.47	10.78	309.67
∑4-rings	13.03	13.16	42.43	10.13	122.92	81.66	41.61	64.02	48.62	10.13	122.92
∑5-rings	5.76	9.82	36.69	8.83	124.36	56.55	34.89	54.26	41.39	5.76	124.36
∑6-rings	2.75	4.35	1.24	3.94	36.57	24.09	15.28	16.37	13.07	1.24	36.57
LMW PAHs	309.67	10.78	30.99	12.97	41.58	49.39	31.03	45.38	66.47	10.78	309.67
HMW PAHs	21.54	27.33	80.35	22.89	283.85	162.29	91.77	134.65	103.09	21.54	283.85
LWM/HMW	14.38	0.39	0.39	0.57	0.15	0.30	0.34	0.34	2.11	0.15	14.38
ANT/(ANT + PHE)	0.13	0.54	0.13	0.14	0.19	0.18	0.22	0.19	0.22	0.13	0.54
FLU/(FLU + PYR)	0.90	0.33	0.34	0.52	0.11	0.26	0.35	0.35	0.39	0.11	0.90
IP/(IP + BghiP)	0.43	0.28	0.78	0.24	0.25	0.13	0.21	0.24	0.32	0.13	0.78
PHN/ANT	6.47	0.87	6.65	6.19	4.17	4.52	3.59	4.23	4.59	0.87	6.65
BaA/CHR	1.00	0.58	0.43	0.79	1.07	0.57	0.94	0.66	0.76	0.43	1.07
BaP/BghiP	1.42	1.06	41.85	0.78	1.90	0.87	1.20	1.53	6.33	0.78	41.85
BaP/BbF	1.09	0.83	0.73	0.59	1.33	0.79	0.97	0.90	0.99	1.09	1.33
BaP/BkF	1.45	1.30	1.19	0.92	1.58	1.19	2.64	1.33	1.46	1.45	1.58
IP/BghiP	0.77	0.40	3.53	0.31	0.33	0.15	0.27	0.32	0.76	0.15	3.53

The literature uses the IP/(IP + BghiP), FLR/(FLR + PYR), and ANT/(ANT + PHN) for source clarification^30–32^. Tables 1, 2 and 3 present the ratios calculated for PAHs, BaA/CHR ratio ranged from 0.76 to 1.13, which is similar to that for coke and coal emission (ratio ranged 1.05–1.17)^33^. The range of BaP/BghiP was 1.30 to 6.33, which indicates that traffic emissions are the most likely cause. Traffic emissions are frequently present when the ratio of BaP/BghiP is greater than 0.60^34^. Additionally, a recorded IP/BghiP ratio greater than 0.4 indicates a diesel engine source. According to Caricchia et al.^35^, the IP/BghiP ratio for petrol engines is around 0.40 and rises to 1.00 for diesel engines. A dominating source of combustion is often considered one with an ANT/(ANT + PHN) ratio > 0.1^30^. All samples had a FLU/(FLU + PYR) ratio below 0.5, indicating petrol emissions, whereas ratios over 0.5 indicated diesel emissions^36^. According to Yunker et al.^30^, the IP/(IP + BghiP) ratio values ranged from 0.27 to 0.33, showing inputs from liquid fossil fuels and/or sources of vehicle combustion, as well as those from the process of burning biomass and coal. Additionally, IP/(IP + BghiP) ratios greater than 0.5 denote coal combustion, whereas IP/(IP + BghiP) ratios of 0.62 denote wood combustion, reflecting the same sources of pollution^30,36^.

### Composition, distribution, and concentration of n-alkanes

The individual *n*-alkanes (C9 to C20) ranged from 0.66 at M12 station to 2417.91 µg/g recorded at M15 station (Table S5 in Supplementary Material). The total *n*-alkane ranged from 448.54 µg/g at M28 location sample to 8442.60 µg/g at M15 location sample, with an average of 1390.10 ±1392.73 µg/g. C-20 recorded the highest average value, while C-10 recorded the lowest value among all the studied stations.

The carbon preference index (CPI) has been applied to *n*-alkanes to help determine their origin. According to Zdanaviciute et al.^37^, the CPI values for petroleum were reported to range from 0.93 to 1.07. On the other hand, according to Bi et al.^15^, the CPI values for plants ranged from 2.3 to 54.3. The greater contribution of *n*-alkanes from manufactured sources, such as burning biomass and petroleum pollution, is indicated by CPI values that are near unity; higher CPI values imply a bigger contribution from natural sources, such as terrestrial vegetation and biogenic^38^. According to Tareq et al.^38^, when the carbon preference index is near 1, it is linked to the emissions from vehicles and other activities; when it is greater than 1, it is linked to terrestrial vegetation. Furthermore, Simoneit^39^ showed that a CPI < 5 has a greater proportion of odd-numbered *n*-alkanes, which are produced by cracking and dehydrating *n*-alkanes and *n*-alcohols, respectively.

The CPI was computed using the subsequent Eq. (1)^40^:

1 $$ {\text{CPI}} = \Sigma \left( {{\text{C9}} - {\text{C19}}} \right)_{{{\text{ODD}}}} /\Sigma \, \left( {{\text{C1}}0 - {\text{C2}}0} \right)_{{{\text{EVEN}}}} , $$

where (Ci–Cj)_ODD_ and (Ci–Cj)_EVEN_ are the residue of the *n*-alkanes with an odd carbon number and that with an even carbon number, respectively, over the range i–j.

The computed CPI values for sediment samples ranged between 0.57 and 3.02 (Table S5 in Supplementary Material). For the sediment, the greatest high CPI values are found in station M22 with CPI > 1. The CPI values above 1.0 indicate that the n-alkanes at these locations may have come from natural sources. CPI levels near 1 are connected to many activities and automobile emissions. In our investigation, the values of CPI larger than 1 demonstrate a preference for *n*-alkanes with odd rather than even carbon numbers. Neither even nor is odd numbered alkane preponderance, which is typical of petroleum and/or mixed sources, represented by CPI values equal to 1^41^.

The *n*-alkanes mean carbon number (MCN) of the sediment samples was computed according to the subsequent Eq. (2):

2 $$ {\text{MCN }} = \Sigma \, \left( {{\text{i }} \times \, \left[ {{\text{C}}_{{\text{i}}} } \right]} \right) \, / \, \left[ {{\text{T}} - {\text{C}}_{{\text{S}}} } \right), $$

where [Ci] and [T–Cs] are the residues of the *n*-alkanes with carbon number i and that of the T-Cs, respectively.

For sediment, the MCN value (Table S5 in Supplementary Material) ranged between 12.12 and 17.94. The high MCN value was at M22 site, while the lower was at M28, with an average MCN value of 14.65 ±1.48. The high relative abundance of trees in comparison to grasses and herbs during the low flow of organic carbon might be the cause of the low MCN values^38^.

### Risk assessment of PAHs

The 10 ng/g tolerance level for BAP has been suggested by the Joint Food and Agriculture Organisation and World Health Organisation Expert Committee on Food Additives, which has examined BAP as a general indication of PAHs^42^. Of the PAHs, BaP is the most hazardous^43^. BAP recorded an average of 15.38-20.91 ng/g above the tolerance limit. A maximum of BaP was recorded in Kharga 109.19 ng/g > Dakhla 102.8 ng/g > Farafra 52.14 ng/g. In addition, the ratios of BaP were above that recorded for BbF, BkF and BghiP in all samples (Tables 1, 2 and 3). The HQ method, which reflects a ratio of the exposure dosage for each PAH to an oral chronic reference dose^44^, was used to assess the risk associated with dietary exposure to non-carcinogenic PAHs. It should be noted that RFD is a threshold dosage or intake that was cautiously selected. Therefore, there is little chance of a negative impact on health if the predicted consumption is lower than the reference dosage (HQ 1). The risk level of 10^–6^ is used by the USEPA^45^, as the threshold at which risk management choices can be made. Decisions on risk management are often based on the cancer risk range of 10^–6^ to 10^–4^. All the investigated PAHs had cancer risk values between 1.43 × 10^–4^ and 1.64 × 10^–1^, as indicated in Table 4. This finding suggests that PAHs in the samples under study pose a moderate risk of cancer. Figure 3 shows the residue amounts of PAH with 3-, 4-, 5-, and 6-rings in the investigated sediment.

**Table 4:** Factorial analysis for residual PAHs (ng/g, dry weight (dw)).

PAHs	ADD	RFD	CSF	HQ	CRI	DI
ACY	0.002	0.02	NA	0.112	ND	2.231
FLU	0.011	0.02	NA	0.536	ND	10.719
PHN	0.027	0.04	NA	0.681	ND	27.258
ANT	0.007	0.3	NA	0.025	ND	7.453
PYR	0.027	0.03	NA	0.889	ND	26.679
BaA	0.016	NA	0.73	ND	1.17E-02	16.004
CHR	0.020	NA	0.007	ND	1.43E-04	20.467
BbF	0.018	NA	0.73	ND	1.34E-02	18.347
BKF	0.011	NA	0.073	ND	8.03E-04	10.999
BaP	0.023	NA	7.3	ND	1.64E-01	22.514
DBA	0.016	NA	7.3	ND	1.15E-01	15.700
IP	0.004	NA	0.73	ND	3.05E-03	4.181
BghiP	0.016	0.04	NA	0.388	ND	15.514
HI				2.631		

*NA* not available, *ND* not determined, *HQ* hazard quotient, *CR* cancer risk index, *ADD* Calculated average daily dose, *CSF* cancer slope factor mg/kg/day, *HI* hazard index, *RFD* reference dose mg/kg/day.

Generally, the ΣPAHs concentrations in the sediments of the three studied areas were lower (10.18 to 790.14, 10.55 to 667.72 and 38.27 to 362.77 ng g^–1^ for Kharga, Dakhla and Farafra Oasis respectively) than El-Mex Bay Alexandria coast, Egypt, (1478–1637 ng g^–1^)^47^ and Abu Qir Bay Alexandria coast, Egypt (69–1464 ng g^–1^)^48^ as shown in Table 5; however, they are similar to those observed in Jiaozhou Bay, China (37.7–290.9 ng g^–1^)^56^ (Table 5).

**Table 5:** PAHs concentration levels in surficial sediments in the Egyptian Mediterranean coast in the present study compared with other coastal studies in the Mediterranean Sea and other world regions.

Study area	Compounds	Average (range) ng/g dw	Ref.
Kharga Oasis, Egypt	Σ13PAHs	158.26 (10.18–790.14)	This study
Dakhla Oasis, Egypt	Σ13PAHs	164.98 (10.55–667.72)	This study
Farafra Oasis, Egypt	Σ13PAHs	180.70 (38.27–362.77)	This study
Egyptian Mediterranean Coast	Σ16PAHs	25,046 (13,156–34,852)	^46^
El-Mex Bay Alexandria coast, Egypt	Σ16PAHs	(1478–1637)	^47^
Abu Qir Bay Alexandria coast, Egypt	Σ16PAHs	(69–1464)	^48^
Egyptian Coast Coastline	Σ16PAHs	(3.5–14,100)	^49^
Egyptian Coastline from	Σ16PAHs	530 (208–1020)	^50^
Mediterranean Coast Napoli (Italy)	Σ16PAHs	(435–872)	^51^
Tunisia, Southern Mediterranean Sea	Σ16PAHs	(175–10,769)	^52^
Milazzo Gulf (Italy) Coastal line	Σ19PAHs	492 (5.6–7402)	^53^
Eastern Basin Mediterranean Sea	Σ24PAHs	(2.2–1056.2)	^54^
French and Spanish coasts	Σ18PAHs	(˃1–8500)	^55^
Jiaozhou Bay, China	–	(37.7–290.9)	^56^
Ulsan Bay, Korea	–	(35–1300)	^57^

## Conclusion

In all of the investigated species, ACE was the predominant congener. The following species' average residue levels (ng/g) of PAHs were found in descending order: The following values are in order of importance: ACE 158.39 > NAP 43.31 > ACY 40.04 > PHN 28.80 > BGP 25.98 > ANT 24.64 > PYR 20.06 > BKF 14.54 > CHY 15.58 > BAA 14.41 > FLT 12.56 > DBA 11.14 > BBF 9.07 > FLU 7.01 > IDP 2.46 > BAP 0.29. The current study's order demonstrates that LMW PAHs were predominant. This tendency may be explained by the decreased solubility of HMW PAHs, which are preferentially absorbed by the digestive systems of biota and adsorbed on particles in bottom sediments. The total *n*-alkane ranged from 448.54 µg/g at M28 location sample to 8442.60 µg/g at M15 location sample, with an average of 1390.10 ±1392.73 µg/g.

### Supplementary Information

Supplementary Tables.

## Data Availability

The corresponding author of the study can provide access to the datasets utilised in this inquiry upon request.
