# Supplementary material for: Distribution, composition and risk assessment of hydrocarbon residue in surficial sediments of El-Dakhla, El-Kharga and El-Farafra oases, Egypt
Source: Sci Rep. 2023 Nov 1;13:18871. doi: 10.1038/s41598-023-46133-9 (PMC10620400; doi:10.1038/s41598-023-46133-9)
Supplement: Supplementary file 1 — Supplementary Tables. [file 41598_2023_46133_MOESM1_ESM.docx]

Supplementary material

**Distribution, composition and risk assessment of hydrocarbon residue in surficial sediments of El-Dakhla, El-Kharga and El-Farafra oases, Egypt**

Tarek O. Said, Safaa Ragab, Amany El Sikaily, Mohamed A. Hassaan, Ahmed El Nemr^*^

**Table S1**. Sediment samples Location.

| **Sample** | **Oasis** | **Temp. (°C)** | **pH** | **E.C.** | **TDS** | **Latitude** | **Longitude** |
| --- | --- | --- | --- | --- | --- | --- | --- |
| M1 | Kharga | 19.4 | 7.28 | 1147 | 734 | 25.42679 | 30.56960 |
| M2 | Kharga | 16.7 | 8.67 | 16060 | 10280 | 25.42083 | 30.57287 |
| M3 | Kharga | 19.7 | 8.49 | 10090 | 6460 | 25.42502 | 30.56691 |
| M4 | Kharga | 21.8 | 8.45 | 10190 | 6520 | 25.42466 | 30.56854 |
| M5 | Kharga | 20.5 | 8.69 | 5560 | 3560 | 25.40109 | 30.59244 |
| M6 | Kharga | 18.6 | 9.22 | 2510 | 1610 | 25.41491 | 30.59682 |
| M7 | Kharga | 34.3 | 6.88 | 475 | 304 | 25.41209 | 30.57864 |
| M8 | Kharga | 28.1 | 6.37 | 2160 | 1384 | 25.40691 | 30.58211 |
| M9 | Kharga | 31.8 | 7.25 | 763 | 488 | 25.37316 | 30.59624 |
| M10 | Kharga | 33 | 7.08 | 657 | 420 | 25.38265 | 30.57260 |
| M11 | Dakhla | 19 | 8.57 | 69700 | 44600 | 25.53500 | 28.97087 |
| M12 | Dakhla | 19.1 | 8.57 | 69800 | 44700 | 25.53500 | 29.05087 |
| M13 | Dakhla | 19 | 8.56 | 70100 | 44800 | 25.53498 | 29.00971 |
| M14 | Dakhla | 20.2 | 8.78 | 94000 | 60200 | 25.53388 | 28.96581 |
| M15 | Dakhla | 19.2 | 8.11 | 31600 | 20200 | 25.54626 | 28.93045 |
| M16 | Dakhla | 18.9 | 8.17 | 27900 | 17850 | 25.54880 | 28.92928 |
| M17 | Dakhla | 15.6 | 8.26 | 12800 | 8190 | 25.53486 | 28.95471 |
| M18 | Dakhla | 15.7 | 8.25 | 87300 | 55900 | 25.54130 | 28.95192 |
| M19 | Dakhla | 15.7 | 8.23 | 82800 | 53000 | 25.53851 | 28.95318 |
| M20 | Dakhla | 16.2 | 8.28 | 84600 | 54100 | 25.53555 | 28.95409 |
| M21 | Dakhla | 16.4 | 8.35 | 81700 | 52300 | 25.53411 | 28.95475 |
| M22 | Dakhla | 16.3 | 8.39 | 84400 | 54000 | 25.52948 | 28.95626 |
| M23 | Dakhla | 16.8 | 8.64 | 11300 | 7250 | 25.54937 | 28.92930 |
| M24 | Dakhla | 18 | 8.41 | 85000 | 54400 | 25.52962 | 28.95580 |
| M25 | Dakhla | 18 | 8.38 | 84600 | 54100 | 25.28800 | 28.95587 |
| M26 | Dakhla | 18.2 | 8.35 | 85200 | 54500 | 25.53190 | 28.95410 |
| M27 | Dakhla | 18.6 | 8.39 | 85100 | 54400 | 25.52980 | 28.95538 |
| M28 | Dakhla | 18.7 | 8.41 | 85000 | 54400 | 25.52242 | 28.96014 |
| M29 | Dakhla | 18 | 8.38 | 85500 | 54700 | 25.52706 | 28.95806 |
| M30 | Dakhla | 18.7 | 8.4 | 86200 | 55600 | 25.51990 | 28.94683 |
| M31 | Dakhla | 18.2 | 8.36 | 85800 | 54900 | 25.52151 | 28.94999 |
| M32 | Dakhla | 18.2 | 8.37 | 86400 | 55300 | 25.52222 | 28.95596 |
| M33 | Farafra | 16.3 | 7.83 | 18075 | 12000 | 27.06750 | 27.89929 |
| M34 | Farafra | 18.6 | 7.62 | 1701 | 1089 | 27.06037 | 27.69921 |
| M35 | Farafra | 18.3 | 7.75 | 1777 | 1138 | 27.06837 | 27.74971 |
| M36 | Farafra | 18.2 | 7.64 | 1823 | 1167 | 27.06837 | 27.79971 |
| M37 | Farafra | 18.3 | 7.76 | 1818 | 1164 | 27.06837 | 27.84971 |
| M38 | Farafra | 18.1 | 7.71 | 1828 | 1170 | 27.06857 | 27.89971 |
| D | Farafra | 20.2 | 7.96 | 494 | 316 | 27.16393 | 27.93134 |
| M | Farafra | 18.3 | 7.74 | 668 | 427 | 27.17026 | 27.93327 |

**Table S2**. The SRM and electron ionization energies used for 13 PAHs.

| **PAH** | **Analytes ID** | **RT (min)** | **Precursor ion mass** | **Product ion mass** | **Collision Energy (eV)** | **Limit of detection (pg)** |
| --- | --- | --- | --- | --- | --- | --- |
| Acenaphthylene | ACY | 8.98 | 152.10 | 151.00 | 20 | 2.5 |
| Fluorene | FLU | 11.07 | 166.00 | 165.00 | 30 | 2.5 |
| Phenanthrene | PHN | 14.60 | 178.00 | 152.00 | 25 | 1.0 |
| Anthracene | ANT | 14.60 | 178.00 | 152.00 | 20 | 1.0 |
| Pyrene | PYR | 20.49 | 202.10 | 201.00 | 20 | 1.0 |
| Benz(a)anthracene | BaA | 26.15 | 228.10 | 226.00 | 35 | 2.5 |
| Chrysene | CHR | 26.15 | 228.10 | 226.00 | 35 | 1.0 |
| Benzo(b)fluoranthene | BbF | 31.00 | 252.10 | 250.00 | 35 | 1.0 |
| Benzo(k)fluoranthene | BkF | 31.00 | 252.10 | 250.00 | 35 | 1.0 |
| Benzo(a)pyrene | BaP | 31.00 | 252.10 | 250.00 | 35 | 1.0 |
| Dibenzo(a.h)anthracene | DahA | 36.30 | 278.10 | 274.10 | 60 | 5.0 |
| Indeno(1.2.3.cd)pyrene | IP | 36.40 | 276.10 | 272.00 | 60 | 5.0 |
| Benzo(g.h.i)perylene | BghiP | 36.40 | 276.10 | 272.00 | 60 | 2.5 |

**Table S3**. The SRM and electron ionization energies used for *n*-alkanes C9 to C20.

| n-alkaine | RT (min) | Precursor ion mass | Product ion mass | Collision Energy (eV) | Limit of detection (ng) |
| --- | --- | --- | --- | --- | --- |
| C-9 | 3.88 | 128.17 | 57.12 | 10 | 2.5 |
| C-10 | 5.07 | 142.18 | 57.12 | 10 | 2.5 |
| C-11 | 6.25 | 156.20 | 57.12 | 10 | 1.0 |
| C-12 | 7.32 | 170.23 | 57.12 | 10 | 1.0 |
| C-13 | 8.32 | 184.23 | 57.12 | 15 | 1.0 |
| C-14 | 9.24 | 198.25 | 57.11 | 15 | 1.0 |
| C-15 | 10.12 | 212.23 | 57.12 | 15 | 1.0 |
| C-16 | 10.94 | 226.29 | 57.11 | 15 | 2.0 |
| C-17 | 11.71 | 240.31 | 57.13 | 15 | 2.0 |
| C-18 | 12.44 | 254.34 | 57.12 | 15 | 2.0 |
| C-19 | 13.14 | 268.34 | 57.12 | 15 | 3.0 |
| C-20 | 13.81 | 282.32 | 57.12 | 15 | 3.0 |

**Table S4a**. Distribution of different concentrations of PAHs residues in Kharga Oasis (ng/g, dry weight (dw)).

| **PAH** | **ACY** | **FLU** | **PHN** | **ANT** | **PYR** | **BaA** | **CHR** | **BbF** | **BKF** | **BaP** | **DBA** | **IP** | **BghiP** | **∑PAHs** |
| --- | --- | --- | --- | --- | --- | --- | --- | --- | --- | --- | --- | --- | --- | --- |
| **M1** | 3.31 | 14.31 | 99.86 | 25.61 | 137.24 | 84.59 | 79.83 | 56.51 | 43.66 | 109.19 | 70.02 | 14.25 | 51.77 | 790.14 |
| **M2** | 0.33 | 4.63 | 25.27 | 8.91 | 29.81 | 21.11 | 18.82 | 17.58 | 7.7 | 25.84 | 15.62 | 4.44 | 12.94 | 192.99 |
| **M3** | 0.25 | 3.54 | 6.42 | 1.07 | 9.39 | 7.83 | 6.88 | 12.5 | 8.92 | 10.68 | 11.64 | 2.68 | 11.23 | 93.03 |
| **M4** | 1.1 | 8.37 | 31.49 | 10.02 | 56.14 | 38.97 | 36.53 | 37.63 | 16.99 | 53.85 | 39.7 | 8.82 | 32.36 | 371.98 |
| **M5** | 0.08 | 3.01 | 3.24 | 2.21 | 6.14 | 2.13 | 2.59 | 4.89 | 2.62 | 2.69 | 3.81 | 1.1 | 11.83 | 46.36 |
| **M6** | 0.2 | 2.87 | 4 | 2.01 | 2.8 | 3.24 | 2.72 | 6.22 | 3.32 | 2.54 | 4.11 | 1.08 | 5.26 | 40.4 |
| **M7** | 0.06 | 1.08 | 1.11 | 1.2 | 0.97 | 0.74 | 0.6 | 0.82 | 0.71 | 0.96 | 0.74 | 0.68 | 0.52 | 10.18 |
| **M8** | 0.05 | 1.01 | 1.18 | 1.36 | 1.29 | 0.79 | 0.67 | 0.97 | 0.78 | 1.07 | 0.88 | 0.81 | 0.72 | 11.6 |
| **M9** | 0.05 | 0.97 | 1.36 | 1.22 | 1.27 | 0.84 | 0.7 | 1.06 | 0.86 | 1.1 | 1.3 | 1.43 | 1.15 | 13.32 |
| **M10** | 0.04 | 1.05 | 1.19 | 1.43 | 1.18 | 0.81 | 0.66 | 0.94 | 0.86 | 1.19 | 1.21 | 1.15 | 0.91 | 12.62 |
| **Av** | 0.55 | 4.08 | 17.51 | 5.5 | 24.62 | 16.1 | 15 | 13.91 | 8.64 | 20.91 | 14.9 | 3.64 | 12.87 | 158.26 |
| **MIN** | 0.04 | 0.97 | 1.11 | 1.07 | 0.97 | 0.74 | 0.6 | 0.82 | 0.71 | 0.96 | 0.74 | 0.68 | 0.52 | 10.18 |
| **MAX** | 3.31 | 14.31 | 99.86 | 25.61 | 137.24 | 84.59 | 79.83 | 56.51 | 43.66 | 109.19 | 70.02 | 14.25 | 51.77 | 790.14 |

**Table S4b**. Distribution of different concentrations of PAHs residues in Dakhla Oasis (ng/g, dry weight (dw)).

| **PAH** | **ACY** | **FLU** | **PHN** | **ANT** | **PYR** | **BaA** | **CHR** | **BbF** | **BKF** | **BaP** | **DBA** | **IP** | **BghiP** | **∑PAHs** |
| --- | --- | --- | --- | --- | --- | --- | --- | --- | --- | --- | --- | --- | --- | --- |
| **M11** | 0.16 | 2.71 | 9.20 | 1.13 | 11.14 | 7.41 | 9.21 | 8.17 | 4.44 | 9.76 | 7.94 | 2.57 | 6.69 | 80.53 |
| **M12** | 2.19 | 15.83 | 39.32 | 9.89 | 41.01 | 18.92 | 25.57 | 26.83 | 13.14 | 23.27 | 0.85 | 1.56 | 21.06 | 239.44 |
| **M13** | 1.7 | 12.61 | 23.92 | 10.00 | 44.57 | 24.02 | 24.73 | 28.88 | 11.41 | 29.41 | 0.52 | 2.08 | 23.63 | 237.48 |
| **M14** | 1.21 | 6.30 | 11.6 | 4.13 | 17.28 | 11.97 | 17.66 | 20.25 | 14.41 | 18.66 | 18.47 | 4.59 | 19.95 | 166.49 |
| **M15** | 1.04 | 8.48 | 51.87 | 9.91 | 47.66 | 21.11 | 26.11 | 22.12 | 17.54 | 24.08 | 20.56 | 5.60 | 38.10 | 294.17 |
| **M16** | 0.39 | 4.27 | 16.84 | 4.02 | 29.78 | 16.02 | 20.31 | 19.78 | 14.68 | 21.99 | 15.45 | 4.05 | 15.94 | 183.5 |
| **M17** | 1.24 | 6.71 | 15.95 | 1.98 | 13.56 | 4.63 | 8.15 | 10.70 | 5.47 | 3.27 | 5.46 | 1.46 | 7.25 | 85.84 |
| **M18** | 0.63 | 6.43 | 15.96 | 4.81 | 20.43 | 14.28 | 18.15 | 20.25 | 7.71 | 20.69 | 16.3 | 4.36 | 15.52 | 165.51 |
| **M19** | 3.75 | 45.34 | 117.36 | 53.30 | 0.38 | 2.08 | 96.69 | 0.52 | 9.14 | 23.67 | 15.68 | 4.88 | 4.94 | 377.74 |
| **M20** | 0.65 | 7.51 | 7.02 | 1.22 | 5.51 | 3.66 | 3.84 | 7.65 | 4.65 | 4.71 | 5.98 | 1.46 | 6.34 | 60.19 |
| **M21** | 0.93 | 8.61 | 58.59 | 14.85 | 107.06 | 65.95 | 66.95 | 72.27 | 31.8 | 102.8 | 68.70 | 17.08 | 52.14 | 667.72 |
| **M22** | 0.32 | 3.66 | 6.44 | 1.90 | 11.87 | 6.71 | 8.27 | 11.41 | 4.67 | 12.96 | 12.20 | 3.03 | 8.02 | 91.46 |
| **M23** | 1.10 | 9.25 | 30.57 | 8.17 | 76.72 | 59.62 | 57.38 | 67.22 | 34.61 | 96.29 | 69.20 | 16.11 | 55.71 | 581.93 |
| **M24** | 0.40 | 5.5 | 6.18 | 1.17 | 8.62 | 2.60 | 4.75 | 4.58 | 2.40 | 1.72 | 2.49 | 1.07 | 3.47 | 44.94 |
| **M25** | 0.53 | 6.47 | 6.27 | 1.21 | 2.80 | 3.02 | 2.66 | 4.90 | 2.70 | 2.19 | 2.91 | 1.17 | 3.20 | 40.02 |
| **M26** | 0.10 | 1.15 | 0.99 | 1.33 | 0.67 | 0.81 | 0.68 | 0.87 | 0.69 | 1.03 | 0.88 | 0.65 | 0.70 | 10.55 |
| **M27** | 0.02 | 1.21 | 1.32 | 1.51 | 0.95 | 0.80 | 0.68 | 0.88 | 0.68 | 0.99 | 0.75 | 0.66 | 0.55 | 11.00 |
| **M28** | 0.09 | 1.14 | 1.08 | 1.42 | 0.76 | 0.79 | 0.66 | 0.92 | 0.72 | 1.04 | 0.80 | 0.76 | 0.71 | 10.89 |
| **M29** | 0.09 | 1.83 | 1.49 | 1.37 | 0.81 | 0.83 | 0.69 | 0.93 | 0.69 | 1.09 | 0.74 | 0.65 | 0.56 | 11.77 |
| **M30** | 2.99 | 4.88 | 9.98 | 1.49 | 9.57 | 4.56 | 7.16 | 6.76 | 5.25 | 8.13 | 7.91 | 2.11 | 9.34 | 80.12 |
| **M31** | 0.94 | 13.05 | 22.14 | 3.39 | 23.24 | 10.98 | 12.58 | 14.17 | 9.96 | 13.61 | 12.11 | 3.00 | 12.3 | 151.47 |
| **M32** | 0.37 | 2.66 | 3.28 | 1.77 | 3.58 | 2.20 | 2.66 | 3.57 | 2.35 | 3.27 | 3.66 | 1.29 | 6.04 | 36.70 |
| **AV** | 0.95 | 7.98 | 20.79 | 6.36 | 21.73 | 12.86 | 18.89 | 16.07 | 9.05 | 19.3 | 13.16 | 3.64 | 14.19 | 164.98 |
| **MIN** | 0.02 | 1.14 | 0.99 | 1.13 | 0.38 | 0.79 | 0.66 | 0.52 | 0.68 | 0.99 | 0.52 | 0.65 | 0.55 | 10.55 |
| **MAX** | 3.75 | 45.34 | 117.36 | 53.3 | 107.06 | 65.95 | 96.69 | 72.27 | 34.61 | 102.8 | 69.2 | 17.08 | 55.71 | 667.72 |

**Table S4c**. Distribution of different concentrations of PAHs residues in Farafra Oasis (ng/g, dry weight (dw)).

| **PAH** | **ACY** | **FLU** | **PHN** | **ANT** | **PYR** | **BaA** | **CHR** | **BbF** | **BKF** | **BaP** | **DBA** | **IP** | **BghiP** | **∑PAHs** |
| --- | --- | --- | --- | --- | --- | --- | --- | --- | --- | --- | --- | --- | --- | --- |
| **D** | 42.91 | 83.32 | 158.89 | 24.55 | 8.94 | 2.05 | 2.05 | 2.02 | 1.52 | 2.21 | 1.95 | 1.19 | 1.55 | 333.16 |
| **M** | 0.16 | 3.40 | 3.35 | 3.86 | 6.80 | 2.34 | 4.02 | 3.97 | 2.55 | 3.30 | 3.19 | 1.23 | 3.12 | 41.30 |
| **M33** | 0.99 | 9.54 | 17.79 | 2.68 | 18.73 | 7.18 | 16.52 | 15.62 | 9.60 | 11.47 | 1.22 | 0.97 | 0.27 | 112.57 |
| **M34** | 0.74 | 4.65 | 6.52 | 1.05 | 4.31 | 2.56 | 3.25 | 3.97 | 2.53 | 2.33 | 2.41 | 0.94 | 2.99 | 38.27 |
| **M35** | 0.57 | 5.98 | 28.25 | 6.77 | 49.25 | 38.03 | 35.64 | 39.29 | 32.93 | 52.14 | 37.34 | 9.15 | 27.42 | 362.77 |
| **M36** | 1.50 | 14.57 | 27.28 | 6.04 | 42.51 | 14.25 | 24.89 | 23.09 | 15.27 | 18.19 | 17.04 | 3.22 | 20.86 | 228.72 |
| **M37** | 0.59 | 8.57 | 17.11 | 4.76 | 16.19 | 12.32 | 13.10 | 14.95 | 5.48 | 14.46 | 10.93 | 3.24 | 12.04 | 133.73 |
| **M38** | 1.22 | 13.81 | 24.55 | 5.81 | 25.85 | 15.17 | 23.00 | 21.02 | 14.28 | 18.96 | 15.06 | 3.98 | 12.39 | 195.08 |
| **Av** | 6.08 | 17.98 | 35.47 | 6.94 | 21.57 | 11.74 | 15.31 | 15.49 | 10.52 | 15.38 | 11.14 | 2.99 | 10.08 | 180.70 |
| **MIN** | 0.16 | 3.40 | 3.35 | 1.05 | 4.31 | 2.05 | 2.05 | 2.02 | 1.52 | 2.21 | 1.22 | 0.94 | 0.27 | 38.27 |
| **MAX** | 42.91 | 83.32 | 158.89 | 24.55 | 49.25 | 38.03 | 35.64 | 39.29 | 32.93 | 52.14 | 37.34 | 9.15 | 27.42 | 362.77 |

**Table S5**. *n*-Alkanes in sediment samples collected from Kharga, Dakhla, and Farafra Oases (µg/g, dry weight (dw)).

|  | **Oasis** | **C9** | **C10** | **C11** | **C12** | **C13** | **C14** | **C15** | **C16** | **C17** | **C18** | **C19** | **C20** | **SUM** | **Average** | **SD** | **CPI** | **MCN** |
| --- | --- | --- | --- | --- | --- | --- | --- | --- | --- | --- | --- | --- | --- | --- | --- | --- | --- | --- |
| **M1** | Kharga | 247.7 | 5.7 | 13.2 | 36.9 | 23.5 | 28.9 | 34.5 | 31.9 | 259.6 | 82.6 | 234.3 | 137.4 | 1136.2 | 94.7 | 98.6 | 2.51 | 15.59 |
| **M2** | Kharga | 247.7 | 1.9 | 13.5 | 29.5 | 14.7 | 15.4 | 17.4 | 11.8 | 57.8 | 21.9 | 36.0 | 49.8 | 517.3 | 43.1 | 66.5 | 2.97 | 12.88 |
| **M3** | Kharga | 246.7 | 2.9 | 16.2 | 34.4 | 17.2 | 20.1 | 13.5 | 12.4 | 27.0 | 31.5 | 59.6 | 79.7 | 561.2 | 46.8 | 66.6 | 2.10 | 13.36 |
| **M4** | Kharga | 244.7 | 2.0 | 16.9 | 39.0 | 26.9 | 34.5 | 24.3 | 36.2 | 70.3 | 54.9 | 86.1 | 112.0 | 747.7 | 62.3 | 65.2 | 1.68 | 14.32 |
| **M5** | Kharga | 246.5 | 3.0 | 13.4 | 29.2 | 16.5 | 19.4 | 16.5 | 21.4 | 41.4 | 48.0 | 82.3 | 100.7 | 638.2 | 53.2 | 67.5 | 1.88 | 14.05 |
| **M6** | Kharga | 246.3 | 4.4 | 10.5 | 23.9 | 14.1 | 15.9 | 12.5 | 13.3 | 21.8 | 26.7 | 39.1 | 65.2 | 493.7 | 41.1 | 66.6 | 2.31 | 12.90 |
| **M7** | Kharga | 247.2 | 3.1 | 17.4 | 38.6 | 17.4 | 21.5 | 10.9 | 13.1 | 16.5 | 26.8 | 40.3 | 62.3 | 515.2 | 42.9 | 66.3 | 2.11 | 12.78 |
| **M8** | Kharga | 247.1 | 3.2 | 14.9 | 33.6 | 16.2 | 17.9 | 11.0 | 12.6 | 19.6 | 30.3 | 41.9 | 67.9 | 516.2 | 43.0 | 66.6 | 2.12 | 12.95 |
| **M9** | Kharga | 246.4 | 3.3 | 15.3 | 32.0 | 15.7 | 19.4 | 14.7 | 19.5 | 22.3 | 29.6 | 37.9 | 55.7 | 511.9 | 42.7 | 65.6 | 2.21 | 12.81 |
| **M10** | Kharga | 247.7 | 4.1 | 13.9 | 33.9 | 17.1 | 18.6 | 11.5 | 12.1 | 18.0 | 26.0 | 42.4 | 67.7 | 513.1 | 42.8 | 66.8 | 2.16 | 12.89 |
| **M11** | Dakhla | 246.8 | 6.5 | 8.6 | 33.2 | 29.4 | 79.2 | 46.7 | 90.7 | 101.1 | 133.9 | 175.1 | 259.0 | 1210.3 | 100.9 | 87.1 | 1.01 | 15.75 |
| **M12** | Dakhla | 246.3 | 0.7 | 45.4 | 127.4 | 110.7 | 258.7 | 117.6 | 493.2 | 677.9 | 451.0 | 635.2 | 585.0 | 3749.1 | 312.4 | 243.6 | 0.96 | 16.64 |
| **M13** | Dakhla | 246.9 | 2.2 | 18.4 | 39.4 | 23.9 | 34.2 | 75.9 | 72.9 | 632.8 | 179.9 | 293.4 | 313.3 | 1933.3 | 161.1 | 186.2 | 2.01 | 16.48 |
| **M14** | Dakhla | 246.2 | 5.6 | 14.5 | 32.5 | 20.2 | 36.9 | 34.7 | 61.5 | 123.1 | 111.1 | 163.1 | 190.6 | 1040.2 | 86.7 | 79.2 | 1.37 | 15.49 |
| **M15** | Dakhla | 247.8 | 4.9 | 10.4 | 36.5 | 64.1 | 191.8 | 370.0 | 703.1 | 1067.1 | 1452.4 | 1876.8 | 2417.9 | 8442.6 | 703.6 | 820.8 | 0.76 | 17.94 |
| **M16** | Dakhla | 247.2 | 2.3 | 11.9 | 28.7 | 17.0 | 22.3 | 39.7 | 40.3 | 254.1 | 130.9 | 188.5 | 208.4 | 1191.2 | 99.3 | 99.3 | 1.75 | 15.88 |
| **M17** | Dakhla | 243.0 | 1.8 | 17.5 | 34.3 | 20.2 | 21.5 | 16.8 | 18.6 | 49.0 | 44.3 | 66.0 | 98.0 | 631.0 | 52.6 | 65.5 | 1.89 | 13.89 |
| **M18** | Dakhla | 244.3 | 3.7 | 21.2 | 49.5 | 35.1 | 93.3 | 56.8 | 117.7 | 150.1 | 217.2 | 314.9 | 426.6 | 1730.5 | 144.2 | 131.8 | 0.91 | 16.49 |
| **M19** | Dakhla | 244.7 | 4.5 | 16.7 | 56.8 | 61.0 | 216.9 | 205.1 | 341.4 | 313.6 | 263.6 | 36.9 | 57.7 | 1819.0 | 151.6 | 124.2 | 0.93 | 15.07 |
| **M20** | Dakhla | 244.5 | 1.9 | 20.0 | 44.6 | 34.6 | 134.1 | 50.2 | 77.6 | 81.3 | 64.7 | 113.0 | 109.2 | 975.9 | 81.3 | 64.7 | 1.26 | 14.53 |
| **M21** | Dakhla | 247.3 | 2.9 | 13.2 | 30.5 | 21.5 | 44.3 | 54.3 | 83.0 | 257.7 | 296.4 | 528.4 | 790.1 | 2369.6 | 197.5 | 245.8 | 0.90 | 17.46 |
| **M22** | Dakhla | 247.2 | 4.5 | 15.8 | 34.0 | 20.6 | 23.2 | 23.8 | 20.2 | 323.0 | 64.5 | 190.6 | 125.9 | 1093.2 | 91.1 | 106.8 | 3.02 | 15.47 |
| **M23** | Dakhla | 246.0 | 0.9 | 20.2 | 62.3 | 53.9 | 201.9 | 72.3 | 228.7 | 106.6 | 110.8 | 122.3 | 137.4 | 1363.2 | 113.6 | 79.0 | 0.84 | 14.92 |
| **M24** | Dakhla | 244.0 | 0.8 | 23.4 | 80.6 | 32.3 | 92.3 | 38.5 | 58.0 | 54.4 | 54.8 | 72.4 | 105.3 | 856.9 | 71.4 | 61.9 | 1.19 | 14.05 |
| **M25** | Dakhla | 247.2 | 2.4 | 21.0 | 54.7 | 39.8 | 129.5 | 69.4 | 89.6 | 61.1 | 54.5 | 56.4 | 80.0 | 905.6 | 75.5 | 63.0 | 1.20 | 13.95 |
| **M26** | Dakhla | 245.3 | 3.1 | 16.0 | 39.2 | 18.3 | 21.6 | 13.6 | 16.6 | 23.1 | 32.7 | 45.1 | 73.3 | 547.8 | 45.7 | 65.5 | 1.94 | 13.14 |
| **M27** | Dakhla | 246.5 | 5.7 | 11.8 | 30.6 | 16.5 | 21.8 | 11.5 | 17.0 | 22.4 | 32.1 | 43.0 | 67.6 | 526.4 | 43.9 | 66.0 | 2.01 | 13.04 |
| **M28** | Dakhla | 248.0 | 6.0 | 13.6 | 30.3 | 13.8 | 14.4 | 9.4 | 9.6 | 14.9 | 19.8 | 27.9 | 41.0 | 448.5 | 37.4 | 67.1 | 2.71 | 12.12 |
| **M29** | Dakhla | 245.4 | 4.1 | 16.3 | 36.4 | 17.2 | 22.3 | 13.2 | 13.3 | 17.4 | 22.0 | 30.4 | 48.9 | 486.9 | 40.6 | 65.6 | 2.31 | 12.45 |
| **M30** | Dakhla | 247.9 | 6.0 | 9.6 | 30.8 | 28.7 | 80.9 | 53.5 | 114.4 | 86.8 | 107.1 | 118.1 | 187.4 | 1071.2 | 89.3 | 72.8 | 1.03 | 15.22 |
| **M31** | Dakhla | 246.1 | 8.6 | 20.0 | 50.3 | 33.9 | 103.5 | 42.4 | 107.9 | 133.5 | 108.9 | 166.9 | 160.4 | 1182.3 | 98.5 | 71.1 | 1.19 | 15.21 |
| **M32** | Dakhla | 247.2 | 4.9 | 15.9 | 37.5 | 23.1 | 39.0 | 22.6 | 50.0 | 38.0 | 46.6 | 61.1 | 85.6 | 671.6 | 56.0 | 63.9 | 1.55 | 13.76 |
| **M33** | Farafra | 246.7 | 5.3 | 18.0 | 41.0 | 32.5 | 101.5 | 125.9 | 272.3 | 898.6 | 345.3 | 474.3 | 457.9 | 3019.1 | 251.6 | 263.9 | 1.47 | 16.80 |
| **M34** | Farafra | 247.3 | 2.7 | 21.6 | 53.6 | 36.1 | 106.3 | 52.8 | 100.7 | 54.7 | 50.7 | 51.8 | 69.5 | 847.7 | 70.6 | 62.7 | 1.21 | 13.81 |
| **M35** | Farafra | 246.5 | 1.7 | 24.5 | 60.0 | 35.2 | 109.1 | 102.8 | 167.8 | 267.6 | 143.4 | 136.7 | 142.0 | 1437.2 | 119.8 | 83.0 | 1.30 | 15.31 |
| **M36** | Farafra | 246.3 | 1.4 | 20.7 | 44.9 | 29.9 | 54.5 | 56.9 | 75.1 | 200.6 | 135.0 | 178.4 | 213.3 | 1256.9 | 104.7 | 85.3 | 1.40 | 15.67 |
| **M37** | Farafra | 247.0 | 2.9 | 24.8 | 66.1 | 70.0 | 311.5 | 137.1 | 292.2 | 145.9 | 148.0 | 151.1 | 203.9 | 1800.7 | 150.1 | 99.8 | 0.76 | 15.23 |
| **M38** | Farafra | 247.3 | 3.0 | 20.3 | 57.7 | 66.3 | 137.7 | 152.6 | 345.5 | 509.1 | 260.2 | 348.2 | 331.8 | 2479.7 | 206.6 | 158.6 | 1.18 | 16.28 |
| **D** | Farafra | 251.8 | 29.8 | 22.6 | 275.2 | 209.8 | 1024.5 | 468.2 | 402.6 | 140.5 | 93.1 | 47.9 | 76.5 | 3042.5 | 253.5 | 282.9 | 0.60 | 14.18 |
| **M** | Farafra | 162.2 | 26.9 | 7.2 | 166.7 | 15.2 | 227.8 | 65.1 | 135.0 | 111.9 | 112.6 | 118.7 | 174.2 | 1323.4 | 110.3 | 69.5 | 0.57 | 15.11 |
